# Factors Associated with Potentially Inappropriate Prescribing in Patients with Prostate Cancer

**DOI:** 10.3390/jcm14030819

**Published:** 2025-01-26

**Authors:** Marija Peulic, Radica Zivkovic Zaric, Milorad Stojadinovic, Miodrag Peulic, Jagoda Gavrilovic, Marija Zivkovic Radojevic, Milos Grujic, Marina Petronijevic, Vladan Mutavdzic, Ognjen Zivkovic, Nevena Randjelovic, Neda Milosavljevic

**Affiliations:** 1 Department of Oncology, Faculty of Medical Sciences, University of Kragujevac, 34000 Kragujevac, Serbia; marija.peulic@gmail.com (M.P.); marinakg2013@gmail.com (M.P.); vmutavdzic@yahoo.com (V.M.); ognjenzivkovic657@gmail.com (O.Z.);; 2 University Clinical Center Kragujevac, 34000 Kragujevac, Serbia; miodrag.peulic@gmail.com (M.P.); jgavrilovic@outlook.com (J.G.); makimarijazivkovic@gmail.com (M.Z.R.); grujicmilos10@gmail.com (M.G.); neda.milosavljevic@yahoo.com (N.M.); 3 Department of Pharmacology and Toxicology, Faculty of Medical Sciences, University of Kragujevac, 34000 Kragujevac, Serbia; 4 Clinic of Nephrology, University Clinical Center of Serbia, 11000 Belgrade, Serbia; dr.milorad.stojadinovic@gmail.com; 5 Department of Surgery, Faculty of Medical Sciences, University of Kragujevac, 34000 Kragujevac, Serbia; 6 Department of Infectious Diseases, Faculty of Medical Sciences, University of Kragujevac, 34000 Kragujevac, Serbia; 7 Department of Clinical Oncology, Faculty of Medical Sciences, University of Kragujevac, 34000 Kragujevac, Serbia

**Keywords:** inappropriate prescribing, prostate cancer, drugs

## Abstract

**Background/Objectives:** Drug prescribing in elderly people with chronic diseases carries certain risks. The desire to treat several different diseases at the same time increases the risk of inadequate drug prescribing. Prostate cancer is a disease of older men and occurs in most men over the age of 65. With age, the risk of prostate cancer increases, but so does the risk of the inadequate prescription of drugs. Our research aimed to highlight the potential inadequate prescription of drugs in patients with prostate cancer, considering that it is mostly a population of older men in whom a greater number of comorbidities is expected, followed by the use of a greater number of drugs. **Methods:** Our investigation was designed as an observational, cross-sectional study of 334 male patients who presented at the Multidisciplinary Tumor Board (MDT) for urological cancers at the University Clinical Center Kragujevac, Kragujevac, Serbia, from 1 September to 15 December 2023. Our primary outcome was obtaining the MAI score. **Results:** Our study showed that a significant number of drugs per patient with a prostate cancer diagnosis were prescribed potentially inadequately. The factors associated with greater risk for PIP were the initial level of PSA, ADT meta (intermittent), and several prescribed drugs; on the other hand, secondary hormonal therapy was the reason for less frequent PIP. **Conclusions:** In conclusion, patients with prostate cancer are under increased risk of inappropriate prescribing when they are prescribed more medication, have high PSA, and have ADT meta (intermittent). To stop the incidence of inappropriate prescribing and its serious economic and health consequences, clinicians should take special care when prescribing new drugs to such patients.

## 1. Introduction

Drug prescribing in elderly individuals with chronic diseases carries certain risks. The desire to treat several different diseases at the same time increases the risk of inadequate drug prescribing [1]. Potentially inappropriate prescribing (PIP) means prescribing drugs that may cause patients more harm than good or not prescribing drugs that are recommended [2]. PIP includes potential inappropriate medication (PIM) and potential prescribing errors (PPOs) [3].

Many factors can contribute to the inadequate prescription of drugs, including patient-related, non-clinical (e.g., age), and clinical (e.g., number of drugs) factors [4]. The analysis of several studies showed that a more significant number of prescribed medications is associated with PIP, as well as a more substantial number of existing comorbidities in the patient, where physical and psychiatric comorbidities have been identified as clinical risk factors [4,5]. The criteria for proving PIP can be explicit and implicit. There are several explicit tools, but one of the most used is the Beers list, last revised in 2019 [6].

Regarding implicit criteria, the medication appropriateness index (MAI) [7] was first developed in the 1990s and was revised twenty years later [8]. It is the most widely used approach. There is a clear association between PIP and adverse drug events, lower quality of life, hospitalizations, and higher healthcare costs [2,9]. The development of new drugs for prostate cancer treatment (such as the second-generation of antiandrogens) carries new challenges [10]. Prostate cancer is a disease of older men and occurs in most men over the age of 65. A Turkish study showed that nearly a third of elderly cancer patients are prescribed potentially inappropriate medication (PIM), which can cause serious drug interactions [11]. 

However, to date, no study has been published that analyzed PIP in patients with prostate carcinoma. Our research aimed to highlight the potential inadequate prescription of drugs in patients with prostate cancer according to the MAI criteria (these criteria detect greater inappropriateness than the explicit criteria [12], considering that it is primarily a population of older men in whom a more significant number of comorbidities is expected, followed by the use of a more substantial number of drugs. Together, these constitute two important risk factors for PIP. By analyzing patients who have prostate cancer (all stages), we investigated factors that could potentially affect PIP and are closely related to the treatment of prostate cancer itself and all types of therapy that this multidisciplinary and complex treatment includes.

## 2. Materials and Methods

Our investigation was designed as an observational, cross-sectional study of 334 male patients who presented at the Multidisciplinary Tumor Board (MDT) for urological cancers at the University Clinical Center Kragujevac (UCCK), Kragujevac, Serbia, from 1 September to 15 December 2023. We selected our patients with the following inclusion criteria: a diagnosis of prostate cancer, older than 18 years with at least one drug prescribed, and signed patient informed consent. The exclusion criteria were the diagnosis of another type of cancer (except prostate cancer), incomplete patient files, dementia, or illiteracy. The study was approved by the Ethics Committee of the UCCK (Number 01/23-268). The study was conducted according to the ethical principles of the World Medical Association of Helsinki for medical research involving human subjects.

The data were collected by examining the patient’s medical history and by interviewing patients with prostate cancer in direct conversation. Our primary outcome was obtaining the MAI score. Potential factors that may have influenced the MAI score were age, body weight, education, smoking habits, coffee drinking, alcohol drinking, drug allergies, side effects, pain, hypertension, angina pectoris, atrial fibrillation, myocardial infarction, heart insufficiency, stroke, dementia, COPD, diabetes mellitus, leukemia, osteoporosis, epilepsy, Parkinson disease, number of hospitalizations, Charles comorbidity score, body temperature, systolic pressure, diastolic pressure, Gleason score, the initial level of PSA (Prostate-Specific Antigen), tumor volume, initial cancer stage, localization of metastasis, time from cancer diagnosis, intervention, radiotherapy, androgen deprivation therapy, secondary hormonal therapy, and number of drugs per patient.

The MAI score was considered using 10 criteria, as described in the original validation study [13]. These were indication, effectiveness, dosage, directions, practicality, drug–drug interactions, drug–disease interaction, pointless duplication, period of therapy, and cost of treatment. An up-to-date platform [14] was used to deliver drug information about the indications of certain drugs and the duration of therapy.

Summary of Product Characteristics (SmPC) were used in the study. For older patients, Beers criteria were also used [6]. Lexicomp was used to check D-type or more severe drug–drug interactions [15]. If prescribed drugs are not covered by National Health Insurance or if the cost of the drug is higher than 10% of its pharmaceutical equivalent, it is also a PIP case.

All collected data were numerically coded, tabulated, and checked for errors. The data were then described by measures of central tendency (if continuous) or by frequencies and relative numbers (percentages). The effects of independent and confounding variables on the study outcome were analyzed using multiple linear regressions. The quality of the regression model was checked by analysis of variance and R square. If the probability of the null hypothesis was 0.05 or below, the results had statistical significance. All calculations were made by Statistical Package for the Social Sciences (SPSS) version 18.0.

## 3. Results

Finally, 334 male patients who fulfilled all of the inclusion criteria were registered in the study. The mean age of the patients was 73.63 ± 6.97 (49–89), and the average weight was 83.59 ± 13.72 kg (54–125 kg). The sociodemographic characteristics of the study sample are shown in Table 1.

All patients included in the study were diagnosed with prostate adenocarcinoma, with the following distribution according to the Gleason score (GS): GS 6 24.7%, GS 7 36.6%, GS 8 22.3%, GS 9 13.1%, and GS 10 3.0%, while the percentage of patients included in the group in which the initial PSA value was over 20 ng/mL was 57.9% (187 patients) (Table 2).

Approximately half of the subjects were initially diagnosed in the non-metastatic stage of the disease (49.5%). In 16% of patients, disease progression (stage IV) was registered during the follow-up period, while 34.4% of the subjects started treatment in the metastatic stage of the disease (Table 2).

From the total number of metastatic patients (including patients who became metastatic as well as patients who were initially diagnosed at stage IV of the disease), 16.4% of patients were in the castration-resistant phase, while a significantly higher percentage (83.6%) belonged to the hormone-sensitive group (Table 2). The highest percentage of respondents at stage IV of the disease had a diagnosis of secondary deposits in bones, as many as 63.9%, while about a quarter of all respondents (25.8%) had metastases in two or more organ systems (bones, lymph nodes, parenchymatous organs) (Table 2).

In 42% of the study population, within the initial multidisciplinary treatment, radiotherapy was not indicated (137 patients). In the group of patients (58%) who underwent radiotherapy, the largest number of them underwent definitive radiotherapy (169 patients), and postoperative RT was carried out in 11 patients—i.e., 3.4%—while salvage RT was indicated in 2.8% of patients (nine patients) (Table 2).

According to the current guidelines for the treatment of prostate cancer for a given stage, androgen deprivation therapy (ADT) was not prescribed in only 16% of patients, while the same was prescribed in the other patients and in the ITT population (intention to treat) at 27.4%. Almost half of the patients received ADT as part of the treatment of metastatic disease (47.9%), while 8.7% of patients, for various reasons, received the same therapy at intermittent intervals.

During their treatment, the patients had up to four therapeutic lines: without any therapy at the time of presentation to the urological MDT—6.0% (19 patients); one therapeutic line—86.5% of 275 patients; 5% (16) of patients underwent two therapeutic lines; and a significantly smaller number of patients had three (2.2%) or four therapeutic lines (0.3%) of patients.

In terms of secondary hormone therapy, as many as 93.9% of patients (a total of 310) included in the study were not treated with secondary hormone therapy, while 15 patients (4.5%) received the same in the pre-cetaxel phase, i.e., five patients (1.5%), after the administration of Docetaxel, were treated with secondary hormone therapy (Table 2).

**Figure 1:**
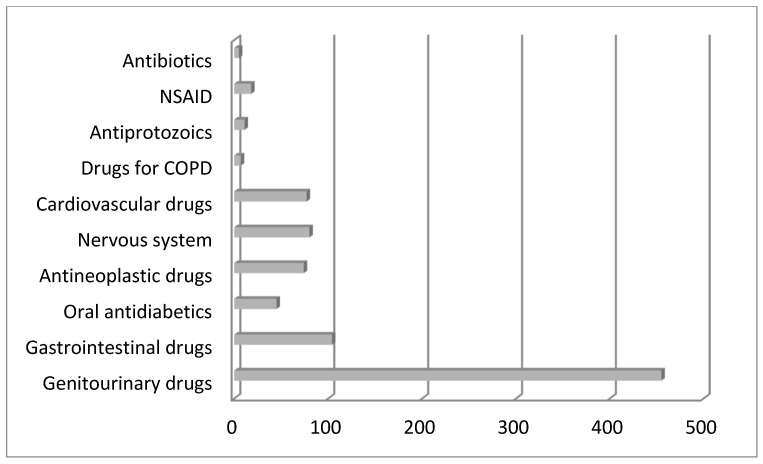
PIP according to MAI criteria related to groups of drugs.

The overall number of prescribed drugs was 1415, with the average per patient being 4.29 ± 2.4. The mean MAI score per patient was two with a range from 0 to 38. The average MAI score per patient was 4.44 ± 6.325. A greater number of patients had 0 (40.5%) MAI scores. A total of 101 out of 1415 (7%) drugs were prescribed without strong indication, and in 105 cases (7%), the risk outweighed the benefit. In 132 (9%) cases of prescribed drugs, the dose was inadequate. Poor duration of prescribed therapy was described in 206 cases (14%). The cost of therapy (a more expensive option was chosen instead of a cheaper therapy) was represented in 231 cases of prescribed drugs (16%). Table 3 and Figure 1 describe the PIP drugs sorted by class and MAI criteria.

We performed multiple linear regressions to analyze the influence of variables on the MAI score. When we entered the factors individually, they stood out as significant: initial level of PSA, phase of cancer, interventions, radiotherapy, ADT for metastasis, secondary hormonal therapy, Charlson comorbidity score, and number of drugs. The final multiple linear regression model is shown in Table 4, with F = 3.2, df1 = 9, df2 = 87, *p* = 0.002, and R^2^= 0.449.

## 4. Discussion

Our study showed that a significant number of drugs per patient with a prostate cancer diagnosis were prescribed potentially inadequately. The factors associated with greater risk for PIP are the initial level of PSA, ADT meta (intermittent), and several prescribed drugs; on the other hand, secondary hormonal therapy is the reason for less frequent PIP.

In our study population, most patients with a mean age of 73.63 ± 6.97 (49–89) finished high school and consumed coffee. This is similar to the general knowledge about the characteristics of patients with prostate cancer [16]. Most new cases are diagnosed in men aged 65 to 74 (38.2%), with a median age at diagnosis of 66 years. One of the causes of the PC was obesity, and the average weight of our patients was 83.59 ± 13.72 kg (54–125 kg). The most commonly reported co-morbid chronic diseases were arterial hypertension, atrial fibrillation, and diabetes mellitus. The literature shows that prostate cancer patients have an average of 0.87 comorbidities and that the likelihood of comorbidities increases with age, with the diagnosis of arterial hypertension being the most frequently recorded [17]. A population-based cohort study conducted by Tiruye et al. showed that the presence of ≥3 comorbidities is associated with poor specific survival [18].

Most of the patients had a Gleason score of 7. The Gleason score is a prognostic factor; namely, patients with a low Gleason score have prolonged cancer-specific survival and vice versa [19]. Our study showed somewhat similar distribution between GS groups, except GS 10 (3.0%), consistent with real-world studies [20]. In comparison, the percentage of patients in PSA over 20 ng/mL group was 57.9%, which also has prognostic value, especially in patients older than 70 [21].

From the total number of metastatic patients, 16.4% were in the castration-resistant phase, while a significantly higher percentage (83.6%) belonged to the hormone-sensitive group. These findings are similar to those in the study conducted by Becker et al. [22]. Steurer et al. reported a 36.8% rate of castration-resistant metastatic prostate cancer patients [23] in a study sample of more than 7000 patients.

The highest percentage of participants in our research had metastasis in the bones, as many as 63.9%, while about a quarter (25.8%) had metastases in two or more organ systems (bones, lymph nodes, parenchymatous organs). Most metastases from prostate cancer occur in the skeletal system due to different biological processes [24], while the presence of metastasis in visceral organs with or without bone involvement shows an unfavorable prognosis [25], and the presence of symptomatic disease requires additional symptomatic and supportive therapy [26].

Radiation therapy (RT) is the most commonly used treatment modality for prostate cancer. Chamie et al., using Surveillance, Epidemiology, and End Results (SEER) data, reported that 57.9% undergo RT for prostate cancer [27]. RT improves overall survival in the prostate, even in the case of metastatic prostate cancer [28]. Our study sample showed that 58% underwent radiotherapy, with most undergoing radiotherapy (169 patients).

LHRH agonist therapy is essential in the treatment of localized and metastatic prostate cancer [29]. Since 1989, ADT has been given intermittently based on PSA level, but it results in a difference in survival compared to continuous application [30]. Given that is the standard of treatment [29], most of our patients received LHRH inhibitors—27.4% of patients in the early stage and 47.9% in metastatic disease. Of those, 8.7% of patients received the therapy at intermittent intervals, which can be explained by the fact that a certain number of patients included in our study were treated many years ago, but also by the fact that they received therapy at smaller institutions, in other centers, that did not provide MDT at the beginning of treatment.

In addition to specific oncological therapy for the primary malignant disease (prostate cancer), the patients included in the study also received therapy prescribed to them by physicians of the appropriate specialties for the treatment of comorbidities. We obtained this information from the patients, their treating physicians, and their families. The patients filled out questionnaires with the help of a study researcher. For their prostate cancer treatment, the patients had up to four therapeutic lines, with most receiving one therapeutic line (86.5%).

In our study sample, the overall number of prescribed drugs was 1415, and the average per patient was 4.29 ± 2.4. According to the WHO core prescribing indicators, Matteiw et al. reported that the average prescribed medication for any oncology patient was 9.63 [31]. There were no data, however, in the available literature about the number of prescribed drugs in prostate cancer patients. Most of the published articles examined concomitant medication while using abiraterone acetate (AA) [32,33,34].

The most commonly prescribed drugs with PIP were alpha-adrenergic receptor blockers, antidiarrheal drugs, 5 alpha-reductase inhibitors, and anxiolytics, while others were much less frequently prescribed. Based on the available literature, the most commonly used concomitant medications in prostate cancer patients were statins, antiplatelet drugs, alpha-blockers, and antibiotics, among others [35].

According to our results, the mean MAI score per patient was 2, ranging from 0 to 38. The average MAI score per patient was 4.44 ± 6.325. This is a significant number, but when we compared our results with those of other studies, it was slightly less; for example, Stojadinovic et al. suggested that the MAI score (± standard deviation) per patient undergoing peritoneal dialysis was 11.7 ± 9.55 [36]. Generally, a higher MAI score is related to poorer quality of life and patient survival [37].

In 7% of the cases, drugs were prescribed without strong indication, and the risk outweighed the benefit in the same percentage of patients. A study conducted by Dumas et al. on breast cancer patients showed that, out of 288 identified medications, 8 were prescribed without strong indication and could affect patient outcomes [38].

In 132 (9%) cases of prescribed drugs, the dose was inadequate in our study. A meta-analysis conducted by Cadogan et al. in 2021 showed that twenty-one studies assessed the appropriateness of prescribing using various tools. The prevalence of patients with ≥1 potentially inappropriate prescription ranged from 15 to 92% [39] in any patient in palliative cancer care. However, there are no data regarding prostate-cancer-specific drug inappropriateness.

A more expensive therapy was chosen instead of a cheaper option in 231 cases (16%). It is known that, according to data from the literature, doctors often prescribe more expensive variants of therapy for various reasons, including specific oncological therapy, the existence of comorbidities, and the availability of drugs at specific times [40].

Further analyses showed that the initial level of PSA (*p* = 0.004), the intermittent administration of ADT (0.045), and the number of prescribed drugs (*p* = 0.006) during docetaxel ADT therapy (0.045) had a significant influence on increasing the MAI score and, thus, elevating the term of inappropriate prescribing in prostate cancer patients. This is logical because higher levels of PSA potentially describe advanced prostate disease with more drugs needed [41]. A higher initial level of PSA can be detected in advanced and metastatic prostate cancer patients [42]. These patients often have symptoms associated with spreading malignant processes, requiring the use of a more significant number of medications, which can potentially interact. Intermittent LHRH inhibitor and docetaxel ADT therapy as factors for inappropriate drug prescribing can also be explained by the fact that this particular therapy is given in a metastatic setting. The prescription of more drugs leads to more potential drug–drug interactions as well as PIP, which is a well-known fact [43]. It was interesting to note that, according to an Italian study, only tobacco use was associated with PSA levels, which should be considered when using PSA-based screening in male smokers [44]. The availability of multiple drugs has changed the treatment course and the natural history of patients with castration-resistant PC, and the administration of numerous consecutive treatments has become very common [45].

Given that the research was conducted in a high-frequency center for the multidisciplinary treatment of prostate cancer and that this type of study was the first to be conducted, we investigated which parameters related to the characteristics of both the patient and the primary tumor or the treatment administered have an influence on inadequate drug prescribing, which was quantified by the MAI score. It should also be mentioned that personalized therapy based on genomic tests is essential for the adequate treatment of patients with prostate cancer [46].

Since a similar study has not been conducted, especially in this study population, we are unable to compare our results with available studies of a similar type. There are only a few reviews in the literature regarding inappropriate drug prescribing in prostate cancer patients. Our work should be considered a valuable basis for similar studies, the results of which will potentially change with new knowledge, but it represents an important basis for further research.

This study has some limitations. The attending physician interviewed each patient, but since some patients were in the day hospital, it is possible that some of the information they gave, along with the medical documentation, was incomplete. Also, this was a single-center study, which could also be a limitation.

## 5. Conclusions

In conclusion, patients with prostate cancer are at an increased risk of inappropriate prescribing when they are prescribed more medication and have high PSA as well as ADT meta (intermittent). To stop the incidence of inappropriate prescribing and its serious economic and health consequences, clinicians should take special care when prescribing new drugs to such patients. Also, electronic systems should be developed to help doctors to minimize PIP in oncology clinics.

## Figures and Tables

**Table 1:** Study population’s sociodemographic characteristics.

Variable	Range; Mean ± SD (Median) or n (%)
Education	
Elementary school	57 (17.1)
High school	237 (70.5)
University degree	37 (13)
Cigarette smoking	56 (16.8)
Alcohol consumption	5 (1.5)
Coffee consumption	280 (83.8)
Allergies	25 (7.5)
Number of specialist who prescribed drugs	3.68 (1.038)
Comorbidities	
Hypertension	233 (69.8)
Atrial fibrillation	64 (19.2)
Myocardial infraction	22 (6.6)
Diabetes mellitus	56 (16.9)
Charlson comorbidity index	0–12 (7.32 ± 2.35)

**Table 2:** Tumor characteristics in study population.

Variable	Frequency	Percent
Gleason score		
GS6	81	24.7
GS7	120	36.6
GS8	73	22.3
GS9	43	13.1
GS10	10	3.0
Initial PSA group		
Less than 10 ng/mL	59	18.3
10–20 ng/mL	77	23.8
More than 20 ng/mL	187	57.9
Initial disease stage		
Non metastatic	164	49.5
Metastatic disease during courses of treatment	53	16
Initial metastatic	114	34.4
Metastatic stage		
mCRPC	25	16.4
Hormone-sensitive mPC	127	83.6
Localization of metastatic disease		
Skeletal	94	63.9
Lymph nodes	11	7.5
Parenchymatous organs	4	2.7
More than one above	38	25.8
Radiation therapy		
Without RT	137	42
Postoperative RT	11	3.4
Salvage RT	9	2.8
Definitive RT	169	51.8
Androgen deprivation therapy (ADT)		
Without ADT	53	16
(Neo)adjuvant	91	27.4
Metastatic setting (continuous)	159	47.9
Metastatic setting (intermittent)	29	8.7
Secondary hormonal therapy (SHT)		
Without SHT	310	93.9
Pre-Docetaxel SHT	15	4.5
Post-Docetaxel SHT	5	1.5

**Table 3:** PIP drugs sorted by class and MAI criteria.

Class of Drugs	Indication	Effectiveness	Dosage	Correct Direct	Practical Direct	DDI	Disease Drug Interaction	Duplication	Duration	Cost
Genitourinary drugs	76	76	79					2	111	111
Gastrointestinal drugs	14	14	22	1					21	32
Oral antidiabetics		2	9	4	1	1	16	1	3	8
Antineoplastic drugs	2		2		2				34	34
Nervous system	5	5		4	11	1	21	2	15	16
Cardiovascular drugs	2	2	8	9	13	3	13	4	8	15
Drugs for COPD		1	1	1	1		2			1
Corticosteroids	1	1	1		1				1	1
Antiprotozoics	1	1	1		1	1	1	1	2	2
Nonsteroidal anti-inflammatory drugs		1	1	1	1	1	2	1	5	5
Antibacterial drugs									5	
Total	101	105	132	22	30	22	58	14	206	231

**Table 4:** The final multiple linear regression model to analyze the impact of variables on the MAI score.

Predictors	B (Cl 95%)	*p* Value
Initial level of PSA	2.055(0.667–3.444)	0.004
ADT meta (intermittent)	7.187 (1.954–12.420)	0.045
Number of prescribed drugs	0.704 (0.211–1.198)	0.006
Secondary hormonal therapy (pre- and post-Docetaxel)	−1.960 (−3.874 to–0.046)	0.045

## Data Availability

Data are unavailable due to privacy reasons.
